# Pediatric emergency care capacity in a low-resource setting: An assessment of district hospitals in Rwanda

**DOI:** 10.1371/journal.pone.0173233

**Published:** 2017-03-03

**Authors:** Celestin Hategeka, Jean Shoveller, Lisine Tuyisenge, Cynthia Kenyon, David F. Cechetto, Larry D. Lynd

**Affiliations:** 1 School of Population and Public Health, Faculty of Medicine, University of British Columbia, Vancouver, British Columbia, Canada; 2 Collaboration for Outcomes Research and Evaluation, Faculty of Pharmaceutical Sciences, University of British Columbia, Vancouver, British Columbia, Canada; 3 Department of Pediatrics, University Teaching Hospital of Kigali, Kigali, Rwanda; 4 Division of Neonatal-Perinatal Medicine; Children's Hospital at London Health Sciences Centre, London, Ontario, Canada; 5 Schulich School of Medicine and Dentistry, Department of Anatomy & Cell Biology, Western University, London, Ontario, Canada; 6 Center for Health Evaluation and Outcome Sciences, Providence Health Research Institute, Vancouver, British Columbia, Canada; Centre Hospitalier Universitaire Vaudois, FRANCE

## Abstract

**Background:**

Health system strengthening is crucial to improving infant and child health outcomes in low-resource countries. While the knowledge related to improving newborn and child survival has advanced remarkably over the past few decades, many healthcare systems in such settings remain unable to effectively deliver pediatric advance life support management. With the introduction of the Emergency Triage, Assessment and Treatment *plus* Admission care (ETAT+)–a locally adapted pediatric advanced life support management program–in Rwandan district hospitals, we undertook this study to assess the extent to which these hospitals are prepared to provide this pediatric advanced life support management. The results of the study will shed light on the resources and support that are currently available to implement ETAT+, which aims to improve care for severely ill infants and children.

**Methods:**

A cross-sectional survey was undertaken in eight district hospitals across Rwanda focusing on the availability of physical and human resources, as well as hospital services organizations to provide emergency triage, assessment and treatment plus admission care for severely ill infants and children.

**Results:**

Many of essential resources deemed necessary for the provision of emergency care for severely ill infants and children were readily available (e.g. drugs and laboratory services). However, only 4/8 hospitals had BVM for newborns; while nebulizer and MDI were not available in 2/8 hospitals. Only 3/8 hospitals had F-75 and ReSoMal. Moreover, there was no adequate triage system across any of the hospitals evaluated. Further, guidelines for neonatal resuscitation and management of malaria were available in 5/8 and in 7/8 hospitals, respectively; while those for child resuscitation and management of sepsis, pneumonia, dehydration and severe malnutrition were available in less than half of the hospitals evaluated.

**Conclusions:**

Our assessment provides evidence to inform new strategies to enhance the capacity of Rwandan district hospitals to provide pediatric advanced life support management. Identifying key gaps in the health care system is required in order to facilitate the implementation and scale up of ETAT+ in Rwanda. These findings also highlight a need to establish an outreach/mentoring program, embedded within the ongoing ETAT+ program, to promote cross-hospital learning exchanges.

## Introduction

Reducing mortality in children younger than five years by two-thirds in developing countries by the year 2015 was a Millennium Development Goal (MDG 4: reducing under-five mortality by two-thirds between 1990 and 2015) adopted by the international community [1]. While Rwanda achieved the MDG 4, the neonatal mortality rate and under-five mortality rate remain high, currently estimated at 20 deaths per 1000 live births and 50 deaths per 1000 live births, respectively [2]. The knowledge needed to improve newborn and child survival has advanced remarkably since the adoption of the MDGs. However, healthcare systems in many low-income countries continue to face many challenges in terms of effectively delivering recommended life -saving interventions for children younger than five years due to limited material and human resources and gaps in knowledge among healthcare professionals working within the health care system [3–6].

District hospitals–the backbone of the healthcare system–are the first-line referral sites in low-income countries like Rwanda. Thus, it is critical that these hospitals have the capacity to provide effective, efficient and high quality care to all patients including severely ill infants and children whose risk of death is greatest in the first 24 hours of admission [3, 7–9]. Accordingly, an intervention to improve emergency triage, assessment and treatment plus admission care (ETAT+) for severely ill infants and children has been implemented in Rwandan district hospitals [10]. Nevertheless, evidence-based interventions are rarely implemented with perfect fidelity under real-world conditions [10, 11]. Therefore, there is a need to understand the capacity of Rwandan district hospitals and identify resource and organizational gaps with respect to healthcare provision to severely ill infants and children in Rwanda. This information can guide further efforts, including the ETAT+ implemenatation, to improve survival of children younger than five years in Rwanda. Thus, the objective of this study was to assess the extent to which Rwandan district hospitals are prepared to provide care for severely ill infants and children, specifically shedding light on the resources and support systems that are available in the district hospitals in Rwanda.

### Healthcare provision in Rwanda

Rwanda has one of the fastest growing populations, currently more than 11 million with a median age of 18.7 years [12]. Rwanda provides healthcare through a mainly publicly funded and operated healthcare system that includes: national referral hospitals, district hospitals, health centers, dispensaries or community health posts, and community health workers [13–15]. Community health workers (CHWs) fulfill important roles in the country’s healthcare system. Generally, the first contact between patients and the formal public health system is the country’s peripheral health units that consist of health centers, dispensaries or community health posts, and CHWs. People also consult private health clinics, pharmacies, and traditional healers. Peripheral health units–staffed by nurses, most of whom have completed the minimum level of nursing training available (A2 level)–provide primary package of health care and are supported by the district hospitals. The district hospitals are generally staffed by nurses mostly with A2 level training, approximately five midwives, and on average, 10 generalist physicians with basic medical training. These district hospitals provide secondary level medical care, including caring for very sick infants and children usually referred from health centers, whilst national referral hospitals provide tertiary specialist care. As of 2012, Rwanda had 41 district hospitals that serve as first-line referral hospitals, and receive all referrals from peripheral health facilities [16]. The Ministry of Health of Rwanda (MOH) sets standards, formulates policy, ensures quality assurance, and is responsible for mobilizing resources and monitoring and evaluation countrywide [14, 15].

## Methods

### Study design and setting

A cross-sectional survey was undertaken to assess district hospital capacity for providing emergency care for severely ill infants and children in Rwanda. The survey was conducted in 2012/2013 during the implementation of the ETAT+ program in Rwandan district hospitals [10], and was carried out in a sample of eight (~20%) district hospitals that were purposely selected out of 41 Rwandan district hospitals to ensure reasonable regional representation. Rwanda has four provinces (East, North, South and West) and the city of Kigali. At least one district hospital in each province and one in the city of Kigali was surveyed, thus resulting in reasonable regional representation. Five of the eight hospitals were located in the Kigali city or a major provincial city, while the remaining three were located in the rural areas.

### Indicators and hospital survey

This assessment of district hospital capacity to provide emergency pediatric and neonatal care included indicators that were selected based on the evidence based clinical practice guidelines as outlined in the MOH adopted “Basic Pediatric Protocols 2011” and previous research in sub-Saharan Africa [17–23]. These indicators focus on the ‘structure’ domain of Donabedian’s framework for measuring quality of healthcare. According to the framework, structure refers to the attributes of the setting in which the care occurs and includes material resources, human resources, and organizational structure [24].

In our assessment, the availability of key resources and hospital organization were assessed by direct observation in the specific district hospital units including the newborn unit, pediatric ward, maternity ward, emergency room, outpatient clinic, and pharmacy, with a standard checklist. These structural indicators were recorded as ‘present’ or ‘absent’ on the survey day.

### Analysis

Availability of key resources was scored using dichotomous indicators (absent or present) for each structural indicator for each hospital, and are presented as frequencies (and proportions) across all district hospitals surveyed.

### Ethics statement

This study received approval from the Research Ethics Board of the University of British Columbia and from the Rwanda Pediatric Association. The current study used existing data that were collected during the implementation of the ETAT+ course in Rwanda, and district hospitals’ information were anonymized and de-identified prior to analysis.

## Results

The current assessment included 8 district hospitals sampled from across Rwanda. The size of these hospitals differed, with the number of pediatric beds per hospital evaluated ranging from 25 to 54 beds and the number of pediatric admission per day per hospital from 4 to 15 admissions (Table 1).

**Table 1:** Hospital size, triage and ward organization.

Hospital size	H1	H2	H3	H4	H5	H6	H7	H8
Number of pediatric beds per hospital	41	54	35	30	30	25	43	40
Number of admission per day per hospital	4	15	5	8	6	10	10	6
Number of ambulance per hospital	3	3	2	3	3	2	2	3
**Triage system**								
Triage and job aids for triage								
Triage system staffed by a trained person at least during the day								
Severely ill children seen in a child specific area								
Specific clinician to immediately attend to very sick children during the day								
Observation area for administering ORS or treating asthma in children’s OPD area								
**Ward organization**								
Separate ward or room for children								
Most seriously ill babies are cared for in a section near nursing station								
Isolation pediatric unit								
Separate admission area for babies born out of the hospital								
Sickest children nursed in direct view of nursing station in the ward								
Children with surgical conditions nursed separate from adults with surgical problems								
Defined area for emergencies								
Mothers of sick newborns facilitated to room in with their babies in the ward								
Kangaroo Mother Care								

Available   Unavailable; ORS, oral rehydration solution; OPD, outpatient department; H1, 4 and 6 are rural located hospitals while the remaining are in urban areas.

### Health human resources

With respect to human resources, three of the eight hospitals had at least one pediatrician. In all hospitals, admissions were done by either a generalist physician (doctor with basic seven years of medical training including one year of internship in a district hospital) or a pediatrician. During the day shift, in nearly all hospitals, there was at least one doctor per pediatric and neonatal department who may leave the hospital after completing ward rounds; while night clinical coverage was done by one generalist doctor covering all hospital wards in three (all rural hospitals) of the eight hospitals, and one doctor covering maternity, pediatric and neonatology in five remaining hospitals. For the day shift, the median number of nurses was 2 (range 1–4) on both general pediatric wards and newborn units. However, while night shift coverage was provided by one dedicated nurse per pediatric ward across all hospitals surveyed, only half of the hospitals had dedicated neonatal unit coverage by one nurse. In the remaining four hospitals, one nurse permanently based in the maternity ward would also attend to patients in the newborn unit.

### Physical resources and organization

#### Emergency triage and ward organization

In all outpatient and emergency departments of the surveyed hospitals, there was neither a functional triage system nor guidelines for triage to help identify and attend immediately to severely ill infants and children, and only one had a specific area with a dedicated physician for severely sick children during the day shift (Table 1). While all the hospitals had newborn and pediatric wards, only three hospitals had organized their wards in a way to allow severely sick newborns (n = 2) and children (n = 1) to be viewed and cared for directly from the nursing station (Table 1). All the hospitals assessed had ambulances for transporting/transferring patients with emergency conditions. Six hospitals had defined areas for emergency care within their newborn and pediatric wards, while seven hospitals had space for Kangaroo Mother Care–a method of care for preterm infants involving infants being carried, usually by the mother, with skin-to-skin contact [25].

#### Guidelines and structured admission record

The availability of updated clinical practice guidelines varied across the surveyed hospitals (Table 2). Guidelines for neonatal resuscitation and management of malaria were available in 5/8 and in 7/8 hospitals, respectively; while those for management of neonatal sepsis, pneumonia, dehydration and severe malnutrition were available in three or fewer hospitals (Table 2). With respect to a structured admission record, a neonatal admission record (NAR) was available in six hospitals and was out of stock in two hospitals; a pediatric admission record (PAR) was not part of care in any institution studied. Neonatal feeding guidelines were readily available but were not consistent within and across hospitals evaluated. Stratification of overall availability of guidelines (Table 2) by hospital location (rural or urban) suggested major rural-urban differences (overall score of 6/24 (25%) and 24/40 (60%) for rural hospitals and urban hospitals respectively).

**Table 2:** Availability of up to date clinical practice guidelines and checklists by district hospital.

Up to date guidelines	H1	H2	H3	H4	H5	H6	H7	H8
Neonatal resuscitation appropriate for clinical area								
Child resuscitation appropriate for clinical area								
Neonatal infection or sepsis								
Management of pneumonia in children								
Management of dehydration in children								
Management of malaria in children								
Management of severe malnutrition (WHO 10 Steps Approach)								
Neonatal feeding								
**Checklist or admission records**								
Checklist for emergency materials								
Neonatal Admission Record (NAR)								
Pediatric admission Record (PAR)								

Available   Unavailable; WHO, World Health Organization; H1, 4 and 6 are rural located hospitals while the remaining are in urban areas.

#### Hygiene and safety

Hygiene and safety measures were considered acceptable in many hospitals. However, while there was limited availability of alcohol hand rub or sanitizer, soap was available in all hospitals. Mosquito nets were available in all hospitals regardless of malaria endemicity of the region, while only five hospitals had a checklist for regularly checking on the availability of emergency drugs in newborn, pediatric and emergency departments (Table 3).

**Table 3:** Hygiene and safety by district hospital.

Hygiene and safety	H1	H2	H3	H4	H5	H6	H7	H8
Sink, clean running water and soap								
Alcohol hand rub or sanitizer								
Sharps put in safety boxes and not overflowing safety boxes								
Lockable area for keeping away dangerous items including drugs								
Toilets clean, dry and accessible to child and /or mother								
All beds in good repair								
All mattresses covered with a Mackintosh, and clean								
All beds covered with mosquito nets *(in malaria prone areas)*								

Available   Unavailable; H1, 4 and 6 are rural located hospitals while the remaining are in urban areas.

#### Laboratory services, equipment, consumables, and drugs

While essential laboratory services were available in all hospitals evaluated, there was limited availability of other laboratory services assessed (Table 4). In terms of specific equipment and consumables, most were available in all hospitals except for intra-osseous (IO) needles, metered dose inhalers (MDI) with spacer, bag valve mask (BVM) for preterm infants and pediatric IV administration sets that were rarely or never stocked in most hospitals (Table 5). Two of the eight hospitals had neither MDI/spacer nor nebulizer. Stratification of availability of resources (e.g., laboratory services, equipment, consumables and drugs) by hospital location (rural or urban) did not suggest any major differences.

**Table 4:** Availability of laboratory and radiology services by district hospital.

Laboratory and radiology services	H1	H2	H3	H4	H5	H6	H7	H8
**Biochemistry**								
Glycaemia ^§^								
Bilirubin								
**Hematology**								
Hemoglobin ^§^								
Full Blood Count								
Sickle cell test								
Cross match & blood bank ^§^								
**Microbiology and Parasitology**								
CSF microscopy & Gram stain ^§^								
Malaria microscopy ^§^								
Stool microscopy								
CSF culture								
Pleural fluid culture								
Joint aspirates culture								
Urine culture								
Blood culture								
**Immunology**								
HIV testing								
**Radiology**								
Plain radiography service ^¶^ ^§^								

Available   Rarely or never available

^§^ Essential laboratory services.

^¶^ Available 7 days/week at least daytime

H1, 4 and 6 are rural located hospitals while the remaining are in urban areas.

**Table 5:** Availability of equipment and consumables by district hospital.

Equipment and consumables	H1	H2	H3	H4	H5	H6	H7	H8
**Oxygen delivery, Monitor, Defibrillator**								
Oxygen (Tank or concentrator)								
Oxygen monitor ^¶^								
BVM for children								
BVM for newborns								
Nasal prongs								
Oxygen mask with reservoir								
CPAP device (bubble CPAP)								
Cardiac monitor/defibrillator								
Automatic External Defibrillator (AED)								
**Suction**								
Suction machine								
Nasogastric tubes								
**Warming**								
Incubator								
**Asthma**								
Nebulizer								
MDI and spacer								
**BP measurement, Infusion and Transfusion**								
Pediatric IV giving sets								
Pediatric cannula								
Blood transfusion sets								
Intra-osseous needle								
Age appropriate BP cuff								
**Phototherapy**								
Phototherapy machine								
**Weighing scale**								
Infants								
Children								

Available   Rarely or never available

^¶^ Not available in all departments—moved from one department to another when needed.

BVM, bag valve mask; MDI, metered dose inhaler; IV, intravenous; BP, blood pressure; CPAP, continuous positive airway pressure

Drugs judged to be essential first-line treatment for the most common conditions, adapted from the WHO essential drug list that was endorsed by the MOH [22], were readily available in most of the hospitals evaluated, and drugs deemed necessary for resuscitation or emergency care were also available in all hospitals (Table 6); however, although adrenaline was available in all hospitals, it was expired in one hospital. Pediatric HAART (highly anti-retrovirus therapy) was available in only 4 of the 8 hospitals (50%). Supportive care medications for severe acute malnutrition including therapeutic milk for severe acute malnutrition (F75) and oral rehydration salts for severely malnourished infants and children (ReSoMal) were available in only 3 of the 8 hospitals (37.5%). Formula for term and preterm babies for short-term supplementation were not available in 5 and 8 hospitals respectively. However, it should be noted that formula for term and preterm babies for short-term supplementation are not on the Rwanda essential drug list [22].

**Table 6:** Availability of drugs included in the pediatric and neonatal standards of hospital care audit tool.

Available in all 8 hospitals	Available in 5–7 hospitals	Available in 2–4 hospitals	Available in 0–1 hospital
**Fluids, antibiotics, HAART & antimalarial**			
Glucose 5%	Chloramphenicol oral	Benzylpenicillin	Half strength Darrows with 5% dextrose *
Ringers lactate and normal saline	Cotrimazole cream/paint	Pediatric HAART	Neonatal ampiclox *
Amoxicillin	Flu/cloxacillin IV		
Chloramphenicol IV			
Flucloxacillin oral			
Ampicillin IV			
Cotromoxazole			
Gentamicin			
Metronidazole IV and oral			
Ciprofloxacin			
Nystatin			
Fluconazole			
Tuberculosis drugs			
Tetracycyline EO			
1^st^ line ACT			
Quininine IV and oral			
**Supportive care**			
Glucose 50% or 10%	Salbutamol inhaled	Potassium oral	Oral morphine
Adrenaline	IV salbutamol	Term formula feeds *†	Preterm formula feeds*†
Diazepam	Iron syrup	Phenorbarbitone IV	Diphtheria antitoxin
Hydrocortisone IV	Zinc supplement	F75	
Ibuprofen	Digoxin	ReSoMal	
Paracetamol	ORS		
Multivitamins, vitamins A and K	Phenobarbitone oral		
IV furosemide			
Mebendazole/Albendazole			
Metoclopramide			
Prednisolone tablets			
F100 or RTUF			
Tetanus prophylaxis			

RTUF, ready to use food; EO, eye ointment; ORS, oral rehydration solution; IV, intra-venous; HIV, Human Immunodeficiency Virus; HAART, Highly anti-retrovirus therapy; ACT, artemisinin based combination therapy; F100 and F75, therapeutic milk for severe malnutrition

* Drugs that are NOT on the Rwandan essential medicine list, thus, explaining their scarce availability in Rwandan health facilities.

^†^ For short term feeding supplementation

## Discussion

The current study aimed to assess the capacity of district hospitals to provide quality care to severely ill infants and children in Rwanda. Our findings suggest that many of the physical resources judged necessary to provide emergency care to very sick children in a typical district hospital in Rwanda were available. In addition, clinical practice guidelines for the management of malaria and neonatal resuscitation were readily available across the district hospitals included in the current assessment. However, gaps remain pertaining to hospital services organization (e.g., non-functional triage system) and limited availability of pediatric clinical practice guidelines pertaining to the leading causes of hospital admission and mortality in children younger than five years in Rwanda (e.g., management of pneumonia, sepsis, dehydration, WHO 10 step approach to management of severe malnutrition).

Consistent with prior research that described inadequate triage for severely ill infants and children in health facilities across low-income countries [3, 4, 26], our assessment points to non-functional triage system along with lack of job aids for triage across all district hospitals surveyed. Further, previous research in such settings has suggested that inadequate emergency, triage, assessment, and treatment of severely ill children is one of the major contributing factors to high hospital mortality rates among children younger than five years within the first 24 to 48 hours of admission [6, 27–29]. A such, an intervention to improve emergency triage, assessment and treatment plus admission care for severely ill infants and children (ETAT+) is currently being implemented in Rwandan district hospitals [10], and we believe that it will help establish a functional triage system in these health facilities and, ultimately, contribute to improvement infant and child health outcomes in Rwanda. For example, training hospital staff in emergency, triage assessment and treatment in Malawi was associated with a reduction in early hospital mortality in children under-five from 47.6 to 37.9 deaths per 1000 admissions after the intervention [26].

Previous studies in low-income countries have consistently described a limited availability of resources to provide emergency care to sick children [3–5]. For example, in a survey of pediatric care conducted in Kenya, English and colleagues found that many essential items for care of severely ill children were lacking in many hospitals [4]. Likewise, shortages of drugs, equipment, and disposable materials made it difficult to implement sepsis management guidelines in resource-constrained settings [5]. While resources may not be as depleted as found in prior studies in other settings, the findings from our survey of district hospital resources also identified major gaps in resource availability (e.g., 50% of hospitals without BVM for newborns or pediatric IV sets, no intra-osseous needles for the management of shock in all hospitals surveyed, no nebulizer or MDI in 25% of the hospitals, and no F-75 and ReSoMal for management of severe acute malnutrition in 5/8 hospitals) that would require improvement in order to enhance the capacity of Rwandan district hospitals to provide emergency care to severely ill infants and children. While basic bacteriology services were readily available across the hospitals surveyed, the lack of bacteriology services including culture potentially leads to empirical treatment without knowledge of bacterial causes of infections, and therefore lack of evidence based prescribing and the development of bacterial resistance. Pediatric HAART were available in only half of the hospitals, perhaps related to stock-outs at the time our survey was conducted.

In Rwanda, previous surveys have highlighted the lack of availability of necessary resources [16, 30]. For example, a survey of quality of care in Rwanda conducted by the Maternal, Newborn, and Child Health in Rwanda (MNCHR) project in 2010 through interviewing healthcare providers, patients and hospital administrators highlighted a lack of physical and human resources as the most important barrier to providing high quality care [30]. Moreover, the 2011 MOH integrated health systems strengthening project mid-term review that focused broadly on structural domain of quality of care delivery found that the main challenges to the health care system in Rwanda included, but were not limited to, insufficient health infrastructure and the use of insufficient and outdated equipment in health facilities [14]. As compared with the current study, previous studies assessed resources *in general*, without special attention to resources required to provide emergency care for children. The current study assessed specifically the availability of resources deemed necessary to provide basic emergency care to severely ill children and infants. Moreover, in the current study, we inventoried whether consumables and drugs were available in wards, whereas previous studies might have relied on self-reports from staff.

Furthermore, in this study, we did not assess whether equipment was outdated, nor did we check whether they were in working order or if the staff knew how to use it. It is important to assess the quality of equipment as poor quality or outdated equipment has been reported in resource-poor settings and might be associated with poor outcomes. For example, Slusher and colleagues found that many phototherapy machines available in sub-Saharan Africa were sub-standard and could not provide enough light to adequately treat neonatal jaundice [31]. Additionally, we found in one facility for example that some drugs (e.g., epinephrine) were available but expired. One of the limitations of our study is also the quantity of physical resources—we just checked whether they were available; however, they might be available but not in sufficient quantities to care for several children. Also, what was not available at the time of the survey was considered not available (out of stock), but it is possible that they were usually available before survey. While using a purposeful sampling approach for selecting the study sample (n = 8 district hospitals) resulted in a reasonable regional representation, this sampling approach might have introduced selection bias that could limit the generalizability of our study findings.

While the current assessment found that there was a limited availability of up to date clinical guidelines in many hospitals that were evaluated, some guidelines that were available were not consistent. For example, treatment (e.g., dosage, frequency) recommendations (e.g., neonatal feeding) varied across the hospitals. Thus, there is a need to harmonize clinical practice guidelines as one of the efforts to improve communication among staff and uptake of these guidelines in Rwanda. Accordingly, continuous medication education (CME) program implementers and policy and decision makers should harmonize CME training in Rwanda. Guidelines of management of malaria were readily available, and this might be explained, at least in part, by existence of malaria-specific programs including malaria surveillance and monitoring. Previous research suggests that use of a standardized admission record (e.g., neonatal (NAR) and pediatric (PAR) admission record) is associated with improved documentation of illness, and that the uptake of these admission records by healthcare professionals in sub-Saharan Africa is quite high [20, 32, 33]. While a NAR was readily available in newborn units, a PAR was not available in any district hospital pediatric wards surveyed. As such, the use of standardized admission records should be recommended in Rwandan district hospitals, especially, given that these hospitals are staffed by healthcare providers with only basic professional training who are generally required to take care of severely ill infants and children without specialist supervision. Nevertheless, efforts are underway to train specialists who will be deploying in all hospitals across the country [34].

In particular, our findings highlight a substantial need to enhance the capacity of Rwandan district hospitals to provide emergency care to severely ill infants and children in the context of the ETAT+ program. Recognizing that not all missing items are necessarily equally important to providing emergency triage, assessment and treatment plus admission care for severely ill infants and children, priority should focus on items that are most needed. First, establishing an adequate triage system across Rwandan district hospitals–this is the focus of the ETAT+ program currently being implemented in Rwanda. Second, efforts should be deployed to ensure appropriate dissemination of clinical practice guidelines endorsed by the Rwanda MoH to district hospitals. Clearly, availing up to date clinical practice guidelines are significantly less expensive to address than what is required to start up bacteriology services. Further, as described earlier, emergency checklist and standardized admission records should be available and their routine use should be enforced in district hospitals. Third, investment in capacity building of health human resources cannot be overemphasized. Multiple efforts are currently underway to train healthcare workers in Rwanda. For example, there is a 7-year partnership between United States and Rwanda to increase the number of healthcare providers including specialist physicians, nurses and midwives [34]. In addition, strengthening CME for in-service healthcare providers is another priority. In response, a 5-year Canadian funded project (Train Support Access Model (TSAM) for Maternal Newborn and Child Health in Rwanda and Burundi) will focus on training in-service healthcare providers across Rwandan district hospitals to increase their competence with regards to management of emergency maternal newborn and child health emergencies.

Fourth, investing in critical resources required to implement ETAT+ in Rwandan district hospitals (e.g., BVM for newborns, IO needles, pediatric giving sets, nebulizer/MDI with spacer). For example, IO access equipment should be readily available in the district hospitals in Rwanda, especially given that dehydration/shock due to diarrheal diseases is one of the leading causes of morbidity and mortality among children younger than five years and the evidence from prior research recommending IO access if IV cannot be promptly established and suggesting that IO access may be ‘easily established’ by healthcare providers with little training and is ‘more rapidly achieved’ than IV access [35, 36]. While IO needles are the most appropriate option to obtain IO access, normal butterfly needles or puncture needles can also be used [17], and are usually widely available in hospitals. Therefore, the lack of IO needles may not hamper successful implementation of ETAT+ in Rwanda. For management of asthma/bronchospasm, 25% of district hospitals lacked both nebulizer and MDI and, therefore efforts should be deployed to have at least one of these items. For spacer, district hospitals can use spacers locally made from plastic bottles that are ubiquitous instead of commercial spacers. This is discussed in the ETAT+ training for healthcare providers in Rwanda. Given that malnutrition is not uncommon, resources for management of severe acute malnutrition in infants and children (e.g., F-75 and ReSoMal) should be available. Alternatively, district hospitals can make F-75 and ReSoMal locally using recommended recipes [17, 18].

In conclusion, our assessment provides evidence on the capacity of Rwandan district hospitals while highlighting gaps in essential resources that can undermine healthcare provision to severely ill infants and children in Rwanda. Identifying these gaps in the Rwandan district health care system is required in order to inform new strategies to facilitate the implementation and scale up on the ETAT+ program, and ultimately, the improvement in infant and child health outcomes in Rwanda. Further, our findings highlight a need to establish an outreach/mentoring program (that could be overseen by the Rwanda Paediatric Association in collaboration with Maternal and Child Health Department of the Rwanda Ministry of Health and other newborn and child health stakeholders) to ensure that adopted clinical practice guidelines are available and used appropriately in these district hospitals, that available equipment is working, that staff know how to use it, and that they do use it. This outreach or mentoring program could be embedded within the ongoing ETAT+ program, to promote cross-hospital learning exchanges.
